# Generation of Multivalent Nanobody-Based Proteins with Improved Neutralization of Long α-Neurotoxins from Elapid Snakes

**DOI:** 10.1021/acs.bioconjchem.2c00220

**Published:** 2022-07-23

**Authors:** Jack Wade, Charlotte Rimbault, Hanif Ali, Line Ledsgaard, Esperanza Rivera-de-Torre, Maher Abou Hachem, Kim Boddum, Nadia Mirza, Markus-Frederik Bohn, Siri A. Sakya, Fulgencio Ruso-Julve, Jan Terje Andersen, Andreas H. Laustsen

**Affiliations:** † Department of Biotechnology and Biomedicine, Technical University of Denmark, DK-2800 Kongens, Lyngby, Denmark; ‡ Quadrucept Bio Ltd., Kemp House, 152 City Road, London EC1V 2NX, United Kingdom; § Fida Biosystems ApS, DK-2860 Søborg, Copenhagen, Denmark; ∥ Sophion Bioscience, DK-2750 Ballerup, Denmark; ⊥ Department of Immunology, Oslo University Hospital Rikshospitalet, N-0372 Oslo, Norway; # Department of Pharmacology, Institute of Clinical Medicine, University of Oslo, N-0372 Oslo, Norway

## Abstract

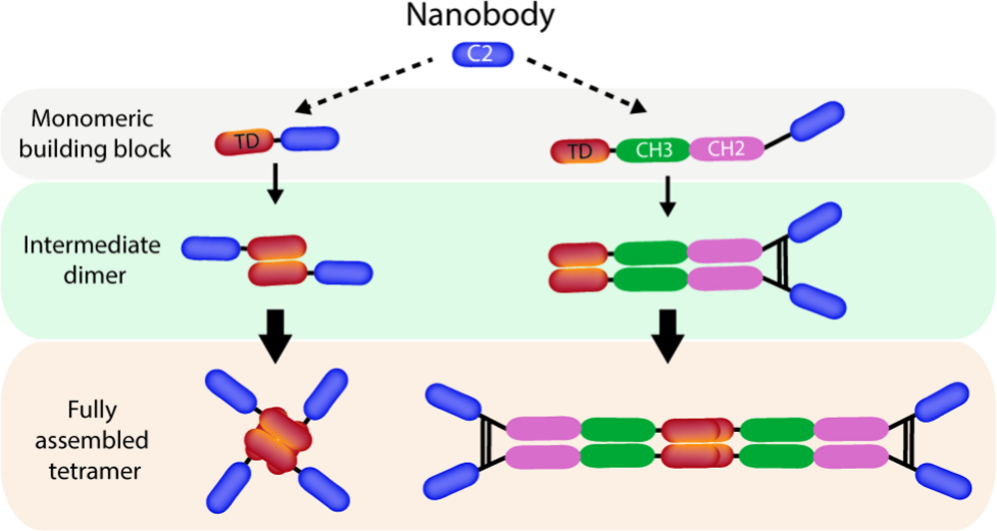

Recombinantly produced biotherapeutics hold promise for improving the current standard of care for snakebite envenoming over conventional serotherapy. Nanobodies have performed well in the clinic, and in the context of antivenom, they have shown the ability to neutralize long α-neurotoxins *in vivo*. Here, we showcase a protein engineering approach to increase the valence and hydrodynamic size of neutralizing nanobodies raised against a long α-neurotoxin (α-cobratoxin) from the venom of the monocled cobra*Naja kaouthia*. Based on the p53 tetramerization domain, a panel of anti-α-cobratoxin nanobody-p53 fusion proteins, termed Quads, were produced with different valences, inclusion or exclusion of Fc regions for endosomal recycling purposes, hydrodynamic sizes, and spatial arrangements, comprising up to 16 binding sites. Measurements of binding affinity and stoichiometry showed that the nanobody binding affinity was retained when incorporated into the Quad scaffold, and all nanobody domains were accessible for toxin binding, subsequently displaying increased blocking potency *in vitro* compared to the monomeric format. Moreover, functional assessment using automated patch-clamp assays demonstrated that the nanobody and Quads displayed neutralizing effects against long α-neurotoxins from both *N. kaouthia* and the forest cobra *N. melanoleuca*. This engineering approach offers a means of altering the valence, endosomal recyclability, and hydrodynamic size of existing nanobody-based therapeutics in a simple plug-and-play fashion and can thus serve as a technology for researchers tailoring therapeutic properties for improved neutralization of soluble targets such as snake toxins.

## Introduction

Snakebite envenoming is a neglected tropical disease with over 2 million victims envenomed each year on a global level. These cases result in more than 100,000 fatalities and 300,000 permanent disabilities,^1^ leaving behind both a large health and socioeconomic burden.^2^ The current standard of care, in the form of antivenom derived from hyperimmunized animals, contains a heterologous polyclonal mixture of both neutralizing and non-neutralizing antibodies.^3^ This form of immunotherapy saves lives and verifies the use of antibodies as a therapeutic approach against envenoming. However, administration of heterologous polyclonal antibodies carries risks of adverse reactions due to the high immunogenicity of the recovered animal-derived antibodies and poor batch-to-batch reproducibility as well as a low therapeutic content of neutralizing antibodies.^1,4^

Recombinantly produced antivenom based on human or humanized antibody sequences could alleviate some of these drawbacks. Further, they can be engineered to have improved therapeutic properties, such as enhanced binding and neutralization potency and optimized pharmacokinetics (PK), depending on what antibody format is employed.^5,6^ As such, different antibody formats have been investigated, of which some have demonstrated good efficacy *in vivo*, including *in vitro* discovered fully human immunoglobulin Gs (IgGs) and nanobodies (V_H_Hs).^7−9^ While nanobodies possess traits desirable for therapeutic development, such as their low immunogenicity^10^ and high thermal stability and production titers in microbial expression systems,^11,12^ they are a monovalent format that experiences rapid clearance, a potential limitation toward their use in certain diseases, including neutralization of toxins with delayed release from the bite site in cases of snakebite envenoming.

Existing approaches to increase the serum half-life of nanobodies involve fusion to proteins, such as IgG Fc or human serum albumin (HSA) that are able to interact with the neonatal Fc receptor (FcRn).^13,14^ FcRn mediates the rescue of IgG from lysosomal degradation through pH-dependent interactions, namely, a higher affinity interaction at acidic relative to neutral pH. Binding with a high affinity within the acidified environment of the endosome prevents trafficking into the lysosome, and a drop in affinity at neutral pH facilitates release back into the serum. Engineering IgG Fc and HSA to have a greater affinity differential between these two pH values has led to the discovery of antibodies and alternative formats with prolonged half-life.^15,16^

The use of self-assembly domains could concomitantly lead to enhanced potency and half-life of nanobodies by increasing valence, accommodating additional nanobody binding and IgG Fc-effector domains that engage both the antigen and FcRn in a single molecule. Larger formats with increased valence could potentially be administered at a lower therapeutic dose, have increased half-life due to a slower rate of glomerular clearance enabling a lowering of the frequency of administration, and have greater exposure to toxins in circulation. The effects of PK on the neutralization of systemically acting toxins for larger, multivalent formats could mechanistically be a benefit in intercepting toxins before they reach their target during the early course of envenoming as well as neutralization of toxins that re-enter circulation in later stages. Investigating the effect of antibody PK on the neutralization of systemically acting toxins has so far received limited attention. However, technologies that allow for the precise tailoring of drug pharmacokinetics without complicating or further adding cost to the manufacturing process might find utility in the development of novel types of recombinant antivenoms with improved therapeutic properties.^17^

In this study, we apply a protein tetramerization technology based on the p53 tetramerization domain (TD) ^18^ to produce a panel of nanobody-based antibody formats with varied hydrodynamic radius, valence, and inclusion or exclusion of IgG Fc domains, termed Quads. Quads rely on intermolecular self-assembly from simple monomeric building blocks to form stable tetramers. As such, we demonstrate successful engineering of Quads with up to 16 binding domains targeting α-cobratoxin (α-cbtx) from *Naja kaouthia*. These novel multivalent molecules were assessed for their long-term structural integrity and binding affinity as well as their neutralization potency and half-life potential. The findings show a functional benefit of increasing valence on blocking and neutralization of α-cbtx, a trait maintained against long α-neurotoxins (LαNtxs) from *N. melanoleuca*, in addition to improved FcRn-mediated recycling and rescue from cellular degradation of Quads designed to contain IgG Fc domains. In combination, the results presented here demonstrate that the Quad multimerization technology could serve as a versatile platform for fine-tuning the molecular parameters of nanobodies, which might find therapeutic utility, for example, in targeting snake toxins like α-cobratoxin with improved efficacy.

## Results and Discussion

### Engineering of Quad Molecules

**Figure 1:**
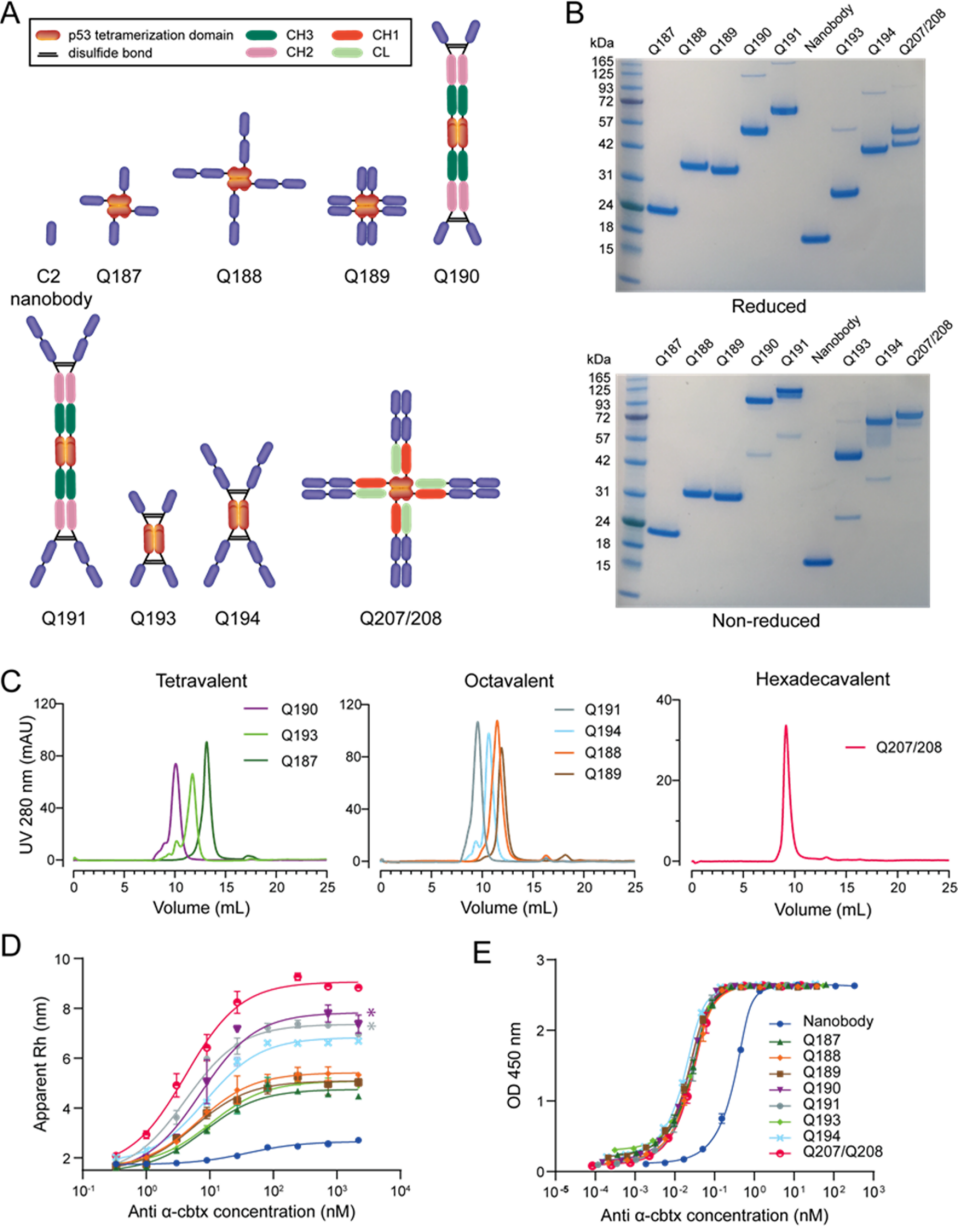
Engineering of Quad molecules. (A) Schematic structural overview of the different Quad formats generated using the p53 tetramerization domain. (B) Nonreducing and reducing colloidal blue-stained SDS-PAGE analysis of the eight Quad constructs and the nanobody. (C) Assessment of purity and monomeric assembly of the Quads via size-exclusion chromatography analysis displayed according to their binding domain valency. The chromatograms were obtained on a HiLoad Superdex200 Increase 10/300 GL column with PBS as an eluent. (D) Binding curve established with FIDA showing the apparent hydrodynamic radius of the indicator α-cbtx-Alexa488 (100 nM) as a function of anti-α-cbtx (0–2.1 μM) in PBST buffer. The *K*_D_ values were calculated from the binding isotherm and are available in Table S2. Represented results are from a single experiment with technical repeats performed in duplicate. *Denotes Quad formats that had increased interaction with the FIDA capillary. (E) ELISA binding assay of Quads to immobilized α-cbtx. Each data point represents the mean of two independent experiments ± SD. The *K*_D_ values were calculated from the binding curves and are available in Table S4.

The low-molecular-weight C2 anti-α-cbtx nanobody was used to engineer novel multivalent antibody formats (Quads) using a flexible multimerization technology described previously.^18^ This yielded a total of eight different multivalent Quads, with or without intact Fc regions, of varying size, shape, and flexibility, possessing valences ranging from tetravalent to hexadecavalent (Figure 1A and Table S1). An important first step in the analysis of these novel multivalent antibody molecules was to show that they could be produced in adequate yields as soluble secreted proteins with high purity and structural integrity. Following transient expression in HEK293 cells and affinity purification of the proteins directly from the culture supernatant, yields of the Quad proteins were calculated (Table 1) using the molar extinction coefficient and protein absorbance at 280 nm. Many of the titers of the Quad proteins with a larger molecular weight were found to be higher than the native nanobody, suggesting that multimerization did not hamper Quad production. Quad titers were also competitive with other tetravalent antibody formats produced in a similar expression system based on a clinically validated IgG scaffold.^19^ The ease of production of Quads from simple monomeric (single polypeptide chain) building blocks that self-assemble into tetramers inside the cell might also indicate that these molecules could potentially be produced microbially, which would provide an opportunity for low-cost manufacture.^20^ If this speculation were to hold true, it would have important implications for the economic feasibility of bringing Quads to the market against neglected tropical diseases, such as snakebite envenoming, where low cost of treatment is essential.^21^

**Table 1:** Blocking Potency, Size-Exclusion Chromatography, and Production Analysis of the Different Quad Formats

	blocking		SEC analysis		production
molecule (valency)	IC_50_ (nM)	V_H_H/Quad	main peak (%)	elution (mL)	yield (mg/L)
V_H_H (1)	0.80	1	100.0	17.56	83
Q187 (4)	0.18	4.7	97.0	13.15	83
Q188 (8)	0.11	7.7	97.0	11.48	75
Q189 (8)	0.10	8.7	95.0	11.90	108
Q190 (4)	0.21	3.9	88.5	10.09	125
Q191 (8)	0.13	6.3	98.0	9.56	65
Q193 (4)	0.19	4.5	81.0	11.75	67
Q194 (8)	0.11	7.4	86.0	10.65	37
Q207/208 (16)	0.05	15.4	98.6	9.17	100

### Structural Analysis of Quad Proteins

To analyze whether the Quads had assembled as multimeric proteins, their size was analyzed under denaturing (SDS-PAGE) and native conditions (size-exclusion chromatography, SEC) (Figure 1B,C). The molecular sizes of the monomeric subunits and fully assembled tetrameric Quads are supplied in Table S1. Single prominent bands on SDS-PAGE separated according to the molecular weight of different Quad proteins. Quads containing disulfide bridges (Q190-Q194 and Q207/208) dissembled into their monomeric subunits when ran under reducing conditions, and there was no obvious proteolytic degradation or aggregation (Figure 1). SEC showed all Quads eluted as single-dominant peaks, except for Q190, Q193, and Q194 that also had a minor percentage of higher molecular weight species, and were >95% tetrameric after being stored for a year at 4 °C (Figure 1C and Table 1 and S6). Quads Q193 and Q194, designed to have a more compact, linear structure, consistently had lower elution volumes and a larger hydrodynamic radius than their Quad counterparts Q187-Q189 that had similar molecular weights but a more globular architecture (Tables 1 and S2). The hydrodynamic radii of the Quads bound to α-cbtx, as determined by flow-induced dispersion analysis (FIDA), ranged from 4.58 to 9.06 nm (Figure 1D and Table S2). A comparison of select unbound Quads Q189, Q193, and Q194 using DLS showed that the population mean sizes ranked in accordance with that seen for the FIDA, and the samples were over 90% monodisperse with no peaks corresponding to partially assembled intermediates (Table S3). The relevance of the increased hydrodynamic size and valence of Quads in comparison to the nanobody could potentially lead to a lower renal clearance^22^ and a more favorable biodistribution profile for toxin neutralization. Furthermore, Quads engineered to contain Fc domains, such as Q190 and Q191, might potentially have an even more prolonged serum half-life, as these Quads contain double the amount of Fc domains compared to a standard IgG antibody. A feature that could potentially be useful when targeting toxins that enter circulation late after the envenoming episode due to venom depot effects.^17^

Drug pharmacokinetics and pharmacodynamics can be influenced by antidrug immune responses. The p53 tetramerization domain is a protein of human origin and was used to drive the assembly of Quads that were either comparable in size to an IgG or smaller than an IgM antibody.^23^ Due to the natural compatibility with the human immune system, it is anticipated that the p53-TD would be less immunogenic than nonhuman tetramerization domains, such as streptavidin and the viral capsid protein VP1. Prediction of immunogenicity is a challenge, and even with fully human antibodies, such as adalimumab, antidrug antibodies have been shown to arise upon administration of the antibody.^24^ The effect of an immune response against human multimerization domains potentially leads to an interference in the biology of the native protein in a patient. In this relation, it could be speculated that because the p53 resides intracellularly, it might cause less of a detriment than tetramerization domains that function within the plasma, such as transthyretin, might do. On the other hand, it cannot be excluded that intracellular domains have more immunogenic properties when entering the extracellular environment, though a comprehensive study conducted by Katchman et al. (2016) mapping p53 immunogenicity did not indicate that the TD domain of p53 is particularly immunogenic.^25^ The structural integrity of the different Quads was further verified in two separate binding assays, either by indirect ELISA or FIDA. Both assays showed that the multivalent Quads were able to bind α-cbtx in a dose-dependent manner. For the ELISA, all Quads exhibited higher binding capacity and lower *K*_D_ values for immobilized α-cbtx compared to the nanobody (Figure 1E and Table S4). Binding to α-cbtx immobilized on a surface promotes avid binding. In the timeframe of this assay, there were, however, no clear differences in binding strength seen between the respective multivalent Quads, likely due to the very low dissociation constants of multiple binding domains simultaneously engaged with the toxins on the surface. Taken together, this confirmed that the Quads were assembled correctly and were functional as tetrameric proteins.

### Quad Molecules Bind Human FcRn in a pH-Dependent Manner and are Rescued from Intracellular Degradation

**Figure 2:**
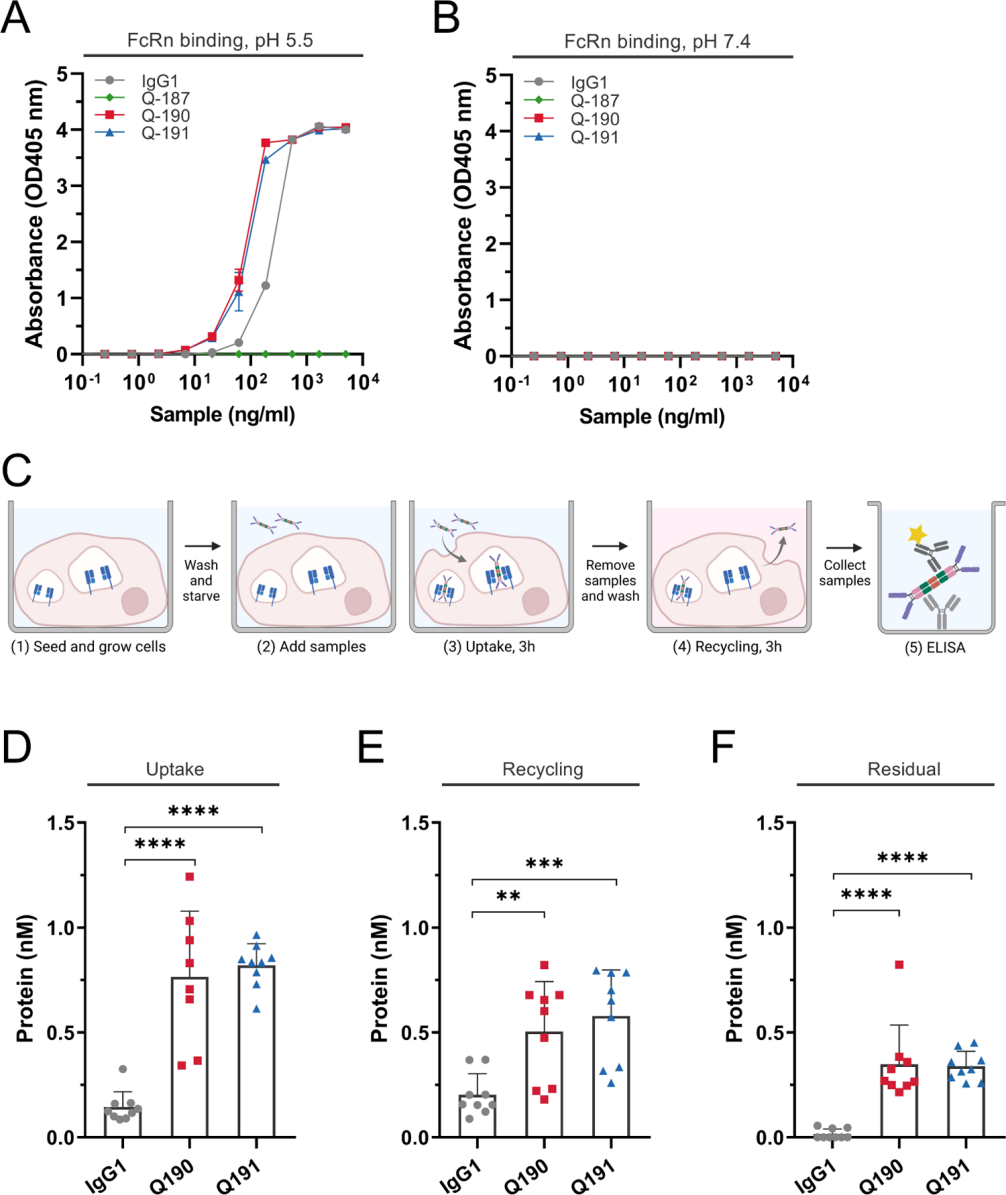
FcRn binding and transport properties of the Quad molecules. (A, B) FcRn-ELISA binding assays were obtained for NIP-IgG1-WT, Q187, Q190, and Q191 at acidic pH (pH 5.5) and neutral pH (pH 7.4). (C) Schematic overview of the HERA protocol. Quads and anti-NIP-IgG1 were added to starved HMEC1-hFcRn cells (1–2) and incubated for 3 h to allow for uptake (3), followed by lysis. Samples were removed, followed by a new 3 h incubation period with fresh medium to allow recycling and release into the medium, or retention inside the cells measured after lysis of the cells (4). Proteins present in the lysates and recycling medium were quantified by two-way anti-Fc ELISA (5). The figure was created with Biorender.com. (D–F) ELISA quantification of the amounts taken up, recycled, or accumulated. Data represents three independent experiments; mean ± SD, unpaired Student’s *t*-test: *p > 0.05, **p > 0.01, ***p > 0.001, **** *p* > 0.0001.

Repurposing FcRn for efficient recycling of Quads requires tight binding at pH <6.0 and low affinity at neutral pH.^26,27^ To verify that the Quads exhibited pH-dependent binding to human FcRn (hFcRn) similar to that of IgG, ELISA was performed. Titrated amounts of the Quads were coated in wells followed by adding a site-specific biotinylated recombinant hFcRn, preincubated with streptavidin conjugated with alkaline phosphatase. The experiment was performed at both pH 5.5 and 7.4, and a full-length human IgG1 with specificity for the hapten NIP was included as a positive control (Figure 2A,B). The results revealed that the Fc-containing Q190 and Q191 bound the receptor at acidic pH, where the binding responses measured were stronger than for the IgG1 control, while none of the formats bound at neutral pH. As expected, Q187 lacking an Fc domain did not bind under either pH conditions.

To address if pH-dependent FcRn binding of the Quads translated into rescue from intracellular degradation, a human endothelial cell-based recycling assay (HERA) based on the adherent human endothelial cell line stably overexpressing human hFcRn (HMEC1-hFcRn) was employed.^13^ Equimolar amounts of Q190 and Q191 were added to the cells in parallel with full-length IgG1. After 3 h of incubation, cells were either lysed to assess the amounts taken up or washed and placed in the IgG-depleted growth medium to allow for cell-internalized molecules to be recycled and released into the medium. After an additional 3 h incubation period, the medium was collected, and the cells were lysed. To quantify the levels of cellular uptake, recycling, and accumulation, samples were analyzed in a two-way Fc-specific ELISA. Data showed that more than fivefold of Q190 and Q191 was detected inside the cells after the uptake step compared to full-length IgG1 (Figure 2D). About 2.5-fold more of the Quads were recycled back to the medium (Figure 2E), while about 7.0-fold more were detected inside the cells at the termination of the assay compared with IgG1 (Figure 2F). Thus, the Quads were found to be exocytosed and released into the medium and, as such, rescued from intracellular degradation, which is in line with pH-dependent hFcRn binding in ELISA. The increased avidity for receptor binding gained through the presence of additional Fc in Quad antibodies may explain the increased uptake, recycling, and accumulation inside cells compared with that of IgG1. To this end, we would expect the Quads to be rescued by hFcRn *in vivo* with a plasma half-life comparable to that of conventional IgG, but further investigation would be required in an *in vivo* setting, such as an hFcRn transgenic mouse model, to define the precise pharmacokinetic parameters of Quad-based antibodies.

In contrast to other self-assembly domains, such as those based on human apoferritin,^28^ the spatial arrangement of the p53 tetramerization product enables the Fc regions to dimerize in the scaffold in a stoichiometry of one Fc to two self-assembly proteins. Although restricted to iterations of two, antigen-binding domains fused to Fc regions affords a clear 1:2 and 1:4 stoichiometry of Fc:antigen-binding domain in a configuration that is accessible to FcRn-mediated recycling, without adding further engineering steps and complexity to the production method.

### Multivalent Quads Show Enhanced Blocking Potency

**Figure 3:**
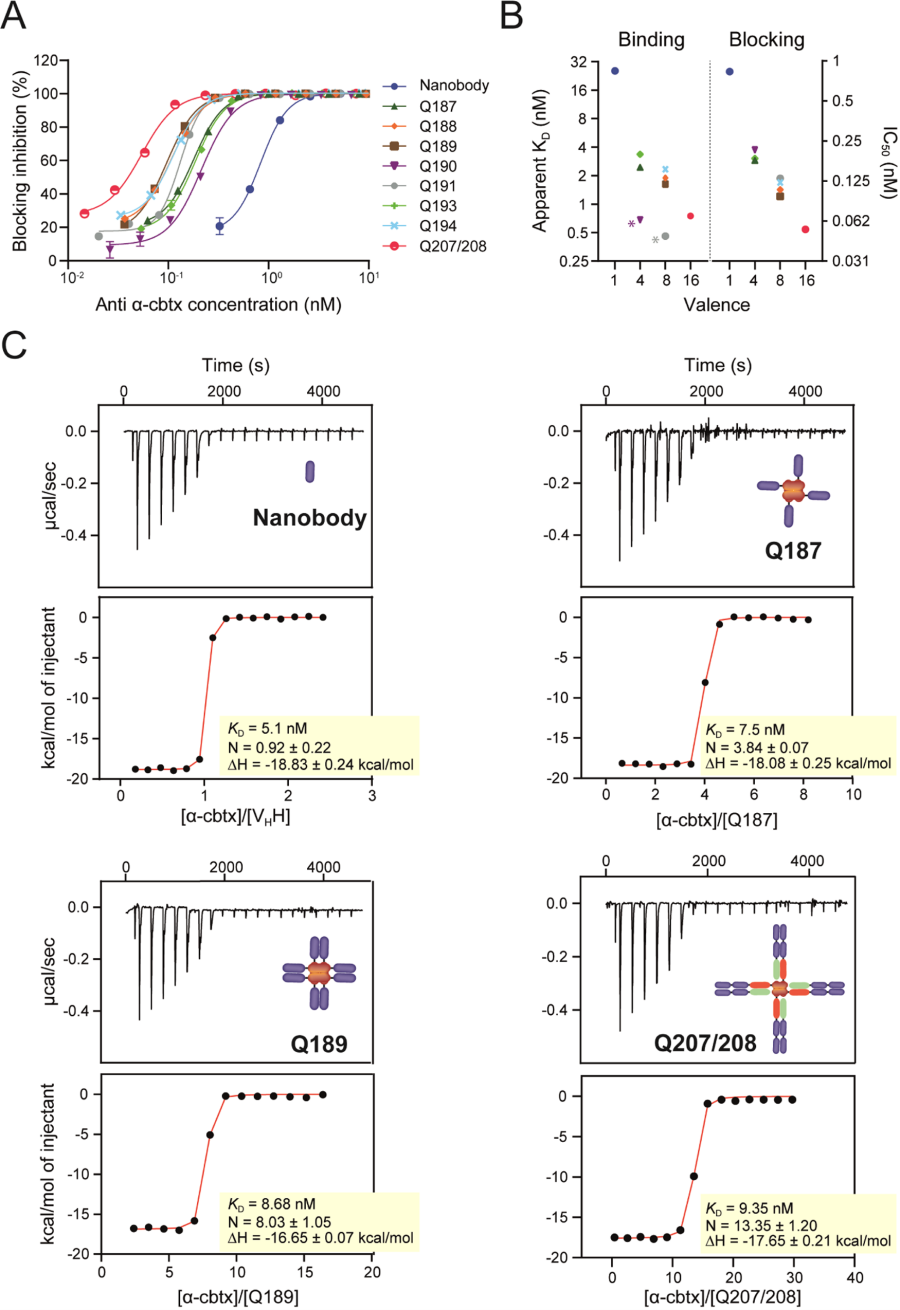
Apparent affinity and blocking characterization of Quad molecules to α-cbtx. (A) Blocking of the α-cbtx/AChR interaction with Quad molecules in an ELISA-based assay. Each data point represents the mean of two independent experiments ± SD. (B) Comparison between apparent *K*_D_ and IC_50_ blocking potency. * Denotes Quad formats that had increased interaction with the FIDA capillary. (C) Representative isothermal titration calorimetry thermograms and curve fits for titrations of V_H_H, Q187, Q189, and Q207/208 into α-cbtx. Binding affinity (*K*_D_) and stoichiometry (N) are the average of two independent titrations.

The C2-nanobody-binding domain used to engineer the different Quad formats has previously been shown to neutralize α-cbtx *in vivo.*(9) In an ELISA-based blocking assay, the nanobody and the different Quad formats were analyzed for their ability to block the interaction between α-cbtx and the acetylcholine receptor (AChR). To assess the effect of increased binding domain valency on the neutralization potency, Quad proteins were directly compared to the monovalent nanobody in the blocking assay. As expected, an increase in neutralization potency was observed for the multivalent Quad proteins compared to the monovalent nanobody (Figure 3A and Table 1), and the IC_50_ and apparent *K*_D_ values measured using FIDA correlated with the increasing binding domain valency (Figure 3B).

Estimating the binding stoichiometry of a representative tetravalent (Q187, *N* = 3.84), octavalent (Q189, *N* = 8.03), and hexadecavalent (Q207/208, *N* = 13.4) Quad by isothermal titration calorimetry indicated that the nanobody domains present in the scaffolds, whether fused adjacently or in tandem to one another, were all accessible to α-cbtx (Figure 2C). Further analysis of thermodynamic parameters verified that binding of the nanobody domains to α-cbtx was only modestly dependent of neighboring nanobody binding domains in the scaffold, with binding enthalpies (16.7–18.1 kcal/mol) and affinities (*K*_D_ = 7–10 nM) for Quads being comparable to the monovalent nanobody (18.8 kcal/mol, 5.1 nM) (Figure 3C and Table S5). The modular design of the nanobody domains within the Quad scaffold opens up the possibility to engineer bispecific or multispecific Quad formats that can neutralize multiple different toxins simultaneously. The use of such multispecific molecules could, thus, reduce the number of components required in a prospective recombinant antivenom product, thereby likely simplifying production and formulation, which could lead to a lower cost of manufacture.

Collectively, these data provide the first proof of concept for the retained nanobody binding affinity and blocking efficacy in multivalent Quad proteins, which offers a strategy for tailoring multispecificities, size, and recycling properties.

### Cross-Neutralization of Structurally Similar Long Neurotoxins Using a Whole-Cell Patch Clamp Assay

**Figure 4:**
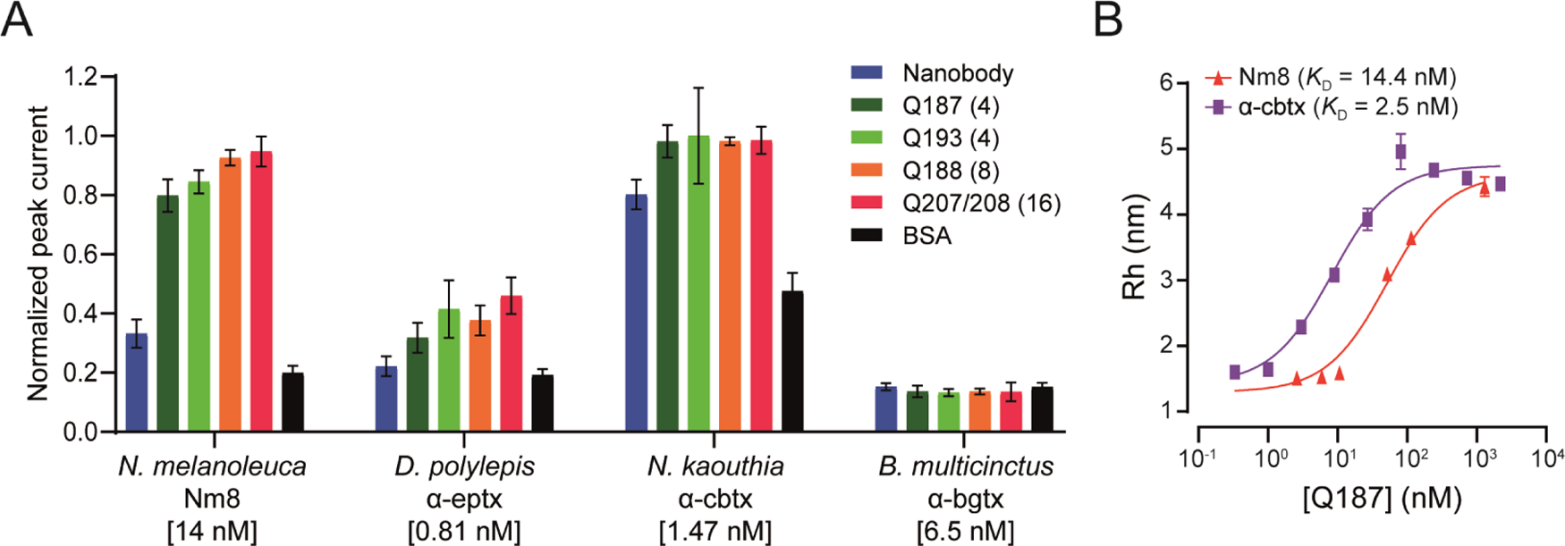
Cross-neutralization of long α-neurotoxins using the C2 nanobody and Quad proteins. (A) Neutralization assessment against α-cbtx from *N. kaouthia,* LαNtxs present in venom fraction Nm8 from *N. melanoleuca*, α-eptx from *D. polylepis,* and α-bgtx from *B. multicinctus*. Error bars represent the ±SD of four replicates. (B) Binding affinity against neutralized LαNtxs characterized using FIDA. Binding profiles were measured as a change in the apparent hydrodynamic radius of the indicators (LαNtxs from *N. kaouthia* and *N. melanoleuca*) following addition of increased concentrations of Q187. The *K*_D_ values were calculated from the binding isotherm. Represented results are from a single experiment with technical repeats performed in duplicate.

The ability of the C2 nanobody and the Quads to functionally neutralize the effects of α-cbtx and three similar LαNtxs was tested *in vitro* using an automated whole-cell patch-clamp assay. Here, a human-derived rhabdomyosarcoma RD cell line, endogenously expressing the muscle-type nicotinic AChR (nAChR), was used to determine the neutralization capacity of the Quads on the current-inhibiting effect elicited by the toxins. The EC_80_ of acetylcholine as well as the IC_80_s of four LαNtxs (α-cbtx from *N. kaouthia*, α-elapitoxin (α-eptx) from *Dendroaspis polylepis*, α-bungarotoxin (α-bgtx) from *Bungarus multicinctus*, and a fraction (Nm8) from *N. melanoleuca* containing an isoform of LαNtx OH55 and long α-neurotoxin 2), were determined. IC_80_ values were 1.47 nM for α-cbtx, 0.81 nM for α-eptx, 6.5 nM for α-bgtx, and 14 nM for Nm8. Neutralization was observed for two out of the four toxins tested, with Nm8 from *N. melanoleuca* venom being neutralized alongside the cognate α-cbtx (Figure 4A). The benefit of increased binding domain valency of Quads, resulting in increased functional affinity, corresponding to low nM apparent *K*_D_ values as determined by FIDA (Figure 4B), can be seen from their ability to fully neutralize both α-cbtx and the LαNtxs present in venom fraction Nm8. Although there was evidence of binding to α-eptx, Quads were unable to achieve full neutralization at the concentrations tested, indicating that the affinity between the Quads and this toxin was possibly too low for neutralization at the tested Quad to toxin ratio. In this relation, it is possible that better neutralization could be achieved using higher Quad concentrations. For α-bgtx, no inhibitory effects were observed, which was not unexpected, as this toxin shares the lowest level of sequence identity relative to α-cbtx (58%), compared to the isoform of LαNtx OH55 (72%) and long α-neurotoxin 2 (83%) from *N. melanoleuca* and α-eptx (79%) from *D. polylepis*. In summary, presenting the neutralizing nanobody in the different Quad formats improves neutralization potency across closely related toxins, suggesting that key interfacial determinants responsible for broad reactivity are maintained within the Quad scaffold.

## Conclusions

In this work, we employed a protein engineering approach to construct tetrameric proteins, termed Quads, comprising a nanobody binding domain and a p53 tetramerization domain, with or without additional Fc domains. These Quad proteins could be engineered to have increased valency of up to 16 binding domains and hydrodynamic radii ranging between 4.58 and 9.06 nm. Importantly, the Quads displayed improved blocking and neutralization *in vitro* and were also able to cross-neutralize LαNtxs from *N. melanoleuca* and, to a lesser degree, neutralize α-eptx from *D. polylepis*. Therefore, this multivalent binding protein concept presents a tunable and versatile technology platform for enhancing the potency of existing nanobodies simply by multimerization, allowing for the assembly of a large number of binding domains in a single molecule. Apart from increasing the molecular size and functional affinity, we proved that the Quads retained pH-dependent binding to hFcRn, which translated into cellular recycling, predictive for half-life extension *in vivo*.^29^ These parameters could potentially be used to further improve the efficacy and pharmacokinetic properties of nanobodies targeting snake toxins. In this relation, one possibility could be to develop Quad molecules with multiple different nanobodies targeting different toxins in a multispecific and multivalent format, enabling that a single molecule could be used to target complex toxin mixtures, i.e., snake venoms. Moreover, the application of Quads outside the context of recombinant antivenom could also find utility in disease settings that rely on avidity for increased safety and potency, such as receptor superclustering, engagement of receptors involved in viral escape and immune regulation, and general multiprotein targeting.

## Methods

### Cloning, Protein Expression, and Purification of Multivalent Anti-α-cobratoxin Quads and Chimeric α7-AChR

The sequence of a high-affinity llama-derived nanobody (C2 V_H_H) against α-cbtx^9^ was used as the binding domain to generate Quads, as previously described.^18,30^ Quad expression plasmids were designed to contain the C2 anti-α-cbtx nanobody sequence linked to either the human p53 tetramerization domain via a flexible linker (G_4_S)_2_ in some configurations or linked to the human IgG1 Fc via the hinge region in other configurations. For those formats containing Fc, the p53-TD domain was linked directly onto the C-terminus of the CH3 domain without any linkers. In configurations where V_H_Hs were linked in tandem, a short linker (G_4_S) was used. A gene encoding a chimeric version of the extracellular domain of α7-acetylcholine receptor (α7-AChR) was also introduced into the pTT5 expression vector.^31^ Genes were all constructed through DNA synthesis (Twist Bioscience), and all constructs contained a C-terminal polyhistidine tag to facilitate purification. Recombinant proteins were generated through transient transfection in HEK293 cells using Expifectamine 293 reagent according to the manufacturer’s recommendations (Thermo Fisher Scientific). Multimerized C2 Quad proteins and recombinant chimeric α7-AChR were purified directly from the culture supernatant using His60 Ni Superflow gravity columns (Clonetech). All proteins were buffer exchanged and concentrated into PBS (137 mM NaCl, 3 mM KCl, 8 mM Na_2_HPO_4_·2H_2_O, 1.4 mM KH_2_PO_4_, pH 7.4) using Amicon columns (Millipore), and aliquots were stored at 4 or −80 °C for long-term storage.

### Size-Exclusion Chromatography

Quad proteins were analyzed at a concentration of 1 mg/mL using an NGC Quest 10 plus chromatography system with a HiLoad Superdex200 increase 10/300 GL with PBS as an eluent. The flow rate used was 0.5 mL/min. The observed size of the proteins was determined for the elution volumes of the main peak after calibration of the column with high-molecular-weight protein standards (protein standard mix 15–600 kDa, 69385, Sigma-Aldrich).

### Toxin Labeling and Biotinylation

Lyophilized α-cbtx (Latoxan, L8114) was labeled with Alexa-Fluor 488 TFP ester as per the manufacturer’s guidelines (Thermo Fisher, 208121). Briefly, the toxin solution (50 μg, 1 mg/mL in PBS) was pH adjusted by adding a tenth volume of 1 M sodium hydroxide. Labeling was performed by adding a twofold molar excess of the dye and incubating at room temperature for 15 min. Free dye was subsequently removed using a dye removal column (Pierce Dye removal column) following the kit instructions, and the presence of free dye was checked using a FIDA One instrument (FIDA Biosystems). Protein concentration was measured using a NanoDrop (Thermo Scientific), and the dye contribution to the absorbance at 280 nm reading was accounted for using equations described in the referenced protocol.

Biotinylation of α-cbtx was performed using EZ-Link NHS-PEG_4_-Biotin at a 1:1.5 (toxin/biotinylation reagent) molar ratio and was purified using Amicon Ultra-4 Centrifugal Filter Units with a 3 kDa MWCO membrane, as previously described.^8^ The degree of biotinylation was analyzed using MALDI-TOF in an Ultraflex II TOF/TOF spectrometer (Bruker Daltonics).

### α-cbtx Binding Analysis by Indirect ELISA

High-binding 96-well plates (Corning) were coated overnight at 4 °C with 50 ng/well of α-cbtx resuspended in PBS. With three washes in between each subsequent step using PBST (137 mM NaCl, 3 mM KCl, 8 mM Na_2_HPO_4_·2H_2_O, 1.4 mM KH_2_PO_4_, 0.1% (v/v) Tween 20) and incubation at room temperature for 1 h, the coated ELISA plates were blocked with PBST + 1% (w/v) BSA (NEB, B9000S), followed by the addition of serially diluted 1 in 3-fold of anti-α-cbtx Quads, starting with a top concentration of 5 μg/mL performed in duplicate. Specific binding of anti-α-cbtx Quads to α-cbtx was detected with the addition of HRP-conjugated anti-His (Abcam, diluted 1:10,000 in PBST), followed by the addition of 100 μL/well of 3,3′,5,5′-tetramethylbenzidine (TMB) to generate the assay signal. The colorimetric reaction was stopped with the addition of 1 M sulfuric acid, and the absorbance was measured at 450 nm using a CLARIOstar microplate reader (BMG Labtech). The dissociation constants were calculated from the curves as described previously,^32^ and presented data points are displayed as mean ± SD values of duplicate measurements.

### FIDA Binding Analysis Instrument Setup

Affinity measurements were conducted on a FIDA One instrument, using light-emitting diode (LED)-induced fluorescence detection (FIDA Biosystems ApS, Copenhagen, Denmark) with an excitation wavelength of 480 nm and a high-pass emission filter (515 nm cut-off). A standard fused-silica capillary (inner diameter: 75 μm, outer diameter: 375 μm, length total: 100 cm, length to detection window: 84 cm, Fida Biosystems ApS) was coated with HS reagent (Fida Biosystems ApS). The capillary was prepared by rinsing with 1 M NaOH for 600 s at 3500 mbar and washed with MilliQ water for 300 s at 3500 mbar. The HS reagent was applied for 600 s at 3500 mbar, followed by a final MilliQ wash, and the baseline was allowed to normalize overnight in water.

### FIDA Binding Characterization of Quad Molecules

Labeled α-cbtx, termed indicator, was diluted to a fixed concentration of 100 nM, and binding was measured as a product of average complex size change over a threefold dilution series (0.11–2,187 nM) of Quad, termed analyte, in PBST (PBS supplemented with 0.05% Tween) buffer. The indicator and analyte were held in separate vials and mixed within the capillary. The template for each assay cycle commenced by equilibrating the capillary with running buffer at 3500 mbar, followed by the sequential injection of analyte and indicator for 20 s at 3500 mbar and 10 s at 50 mbar, respectively. Mobilization of indicator and analyte toward the detector was initiated with a final injection of analyte for 180 s at 400 mbar. To improve diffusivity of larger Quads, a mobilization time of 430 s at 167 mbar was applied, and for Quads that interacted more strongly with the capillary, a wash step consisting of 300 s at 3500 mbar using 1 M NaCl and 1% Tween was used. Each signal (Taylorgram) was processed in the FIDA One data analysis software (V2.04), whereby the change in diffusivity following binding was converted into the hydrodynamic radius using equations described previously.^33^ The Taylorgram fraction was adjusted manually to ensure that there was a sufficient baseline to ensure accurate fitting, and a minimal fitting fraction was employed. A mean data point of duplicate measurements of the hydrodynamic radius for each analyte concentration was plotted on a log10 scale in FIDA analysis software, and a 1:1 toxin/Quad binding stoichiometry and an excess indicator model were used to fit the measurements. The *K*_D_ values were calculated directly from the binding isotherm fits.^33^ Proteins and running buffer were kept in separate compartment chambers at 4 °C and room temperature, respectively. Technical repeats for each Quad were performed at least to the duplicate level at a capillary temperature of 25 °C.

### Dynamic Light Scattering (DLS)

The Dh of the Quads was determined by dynamic light scattering using a ZETASIZER NANO (Malvern) instrument. Quad protein (1 mg/mL, PBS) was spun at a maximum speed for 10 min at 4 °C and added to a 1 mL cuvette (LabX, DTS0012) and measured at a fixed temperature of 20 °C with a duration of 10 s per read. Particle size determinations were obtained from an accumulation of three reads using the instrument software.

### Isothermal Titration Calorimetry (ITC)

The binding affinities between α-cbtx and the C2 nanobody and selected Quad molecules were analyzed by ITC, using a MicroCal ITC_200_ instrument (Malvern Panalytical) at 25 °C. Proteins were dialyzed (Thermo Scientific Slide-A-Lyzer Dialysis Cassettes, 3.5 K MWCO, 0.5 mL: 11859410) against 250 volumes of sterile PBS, followed by centrifugation at max speed at 4 °C for 20 min. Thereafter, protein concentrations were determined using the theoretical molar extinction coefficients calculated based on the amino acid content using the Expasy ProtParam tool. The C2 nanobody and the Quads were loaded in the cell and titrated with the toxin, and the nanobody was diluted to 30 μM in the cell and the toxin to 300 μM in the syringe. For the Quads with 4, 8, and 16 binding sites, the toxin concentration was fixed to 220 μM and Quads diluted to 4, 2, and 1 μM, respectively. The titrations were carried out in two independent duplicates using different toxin batches at 25 °C, starting with an injection of 0.4 μL followed by 19 injections of 2.0 μL (subsequently spaced by 240 s between the injections). After correction with the heat of dilution, as determined by blank injections of toxins into a buffer using the same injection regiment as for the toxin-protein titrations, the thermograms were integrated. A single set of equivalent and independent binding site model was fit to the resulting binding isotherms, which allowed for the determination of the equilibrium association constant (*K*_A_), the binding stoichiometry (N), and the molar binding enthalpy (Δ*H*). The data are reported as the mean ± SD of duplicate measurements, and data processing and model fits were performed using the Origin plug-in provided with the instrument.

### FcRn Binding ELISA

ELISA was performed to quantify the FcRn binding of the Quad molecules in a pH-dependent manner. Quads (Q187, Q190, and Q191) and control (full-length IgG1 WT) were designed, produced, and purified as described previously.^13,34−36^ Molecules were diluted in PBS at a final dilution ranging from 0.488 to 1,000 ng/mL and coated by adding 100 μL to ELISA wells and incubated at 4 °C overnight. Plates were blocked by adding 250 μL of PBS supplemented with 4% (w/v) skimmed milk (M; VWR, A0830), followed by incubation on a shaker for 1 h at room temperature. Plates were washed four times with 200 μL of PBST between all subsequent steps. Next, biotinylated truncated monomeric hFcRn (hFcRn-bio) (Immunitrack, ITF01) was incubated with streptavidin conjugated with alkaline phosphatase (Roche, 11089161001) at a 1:1 molar ratio for 20 min and added to the plate at final concentrations of 0.25 μg/mL FcRn and 3.36 μg/mL streptavidin-AP diluted in PBST-M (pH 5.5 and 7.4, respectively). After 1 h, the ELISA signal was developed by adding 100 μL of 10 μg/mL *p*-nitrophenyl-phosphate substrate (Sigma-Aldrich, S0942-200TAB) dissolved in diethanolamine solution to all wells. A Sunrise spectrophotometer (Tecan) was used to measure absorbance at 405 nm.

### Human Endothelial Cell Line Stably Overexpressing hFcRn

HMEC-1 cells stably expressing HA-FcRn-EGFP (HMEC-1-FcRn)^37^ were cultured at 37 °C and 8% CO_2_ in MCDB131 medium (Gibco, 10372019) supplemented with 2 mM l-glutamine (Sigma, G4251), 25 μg/mL streptomycin/25 U/mL penicillin (Sigma-Aldrich, P4458), 10% FCS (Sigma-Aldrich, F7524), 10 ng/mL mouse epidermal growth factor (Gibco, PMG8043), 1 μg/mL hydrocortisone (Sigma-Aldrich H0888), 100 μg/mL G418 (Gibco, 11558616), and 50 μg/mL blasticidine (Gibco, A1113903) to maintain FcRn expression.

### HERA

HERA experiments were performed as described in Grevys et al., 2018.^13^ Briefly, 1.5 × 10^5^ HMEC-1-FcRn cells were seeded in 250 μL of culturing medium per well in two 48-well plates (Costar) (uptake and recycling plate). The medium was removed from all wells 20–24 h after seeding, and the cells were washed twice in 250 μL of prewarmed Hank’s balanced salt solution (HBSS; Thermo Fisher, 14025100). Cells were starved at 37 °C for 1 h in the prewarmed HBSS. Next, anti-NIP-IgG1 and Quad molecules (Q190 and Q191) were prepared at a final concentration of 800 nM in the prewarmed HBSS and added to cells at a final volume of 125 μL in technical triplicates in both plates. After a 3 h incubation period, the samples were removed, and the cells were washed four times in 250 μL ice-cold HBSS. Uptake plates were frozen at −80 °C following aspiration of washing medium, while 220 μL of prewarmed serum-free growth medium supplemented with 1X MEM nonessential amino acids (Gibco, 11140-050) was added to the recycling plates. After another 3 h incubation period, recycling samples were harvested and frozen at −20 °C. Residual plates were washed four times with ice-cold HBSS and frozen at −80 °C to the day of analysis.

For HERA analysis, frozen cells were lysed by adding 220 μL of RIPA buffer (Thermo Fisher, 89901) supplemented with 1X complete protease inhibitor cocktail (Roche, 11836145001) and incubated on a shaker for 10 min on ice. Cellular debris were removed by 5 min centrifugation at 10,000*g*. Proteins present in the lysates and recycling medium were quantified by two-way anti-Fc ELISA. Ninety-six well plates (Costar) were coated with anti-hIgG Fc (Sigma, I2136) diluted 1:1,000 in PBS and incubated overnight at 4 °C. The next day, plates were blocked by adding 250 μL of PBST-M and washed four times with PBST. Next, cell lysates (containing uptake and residual) and medium (containing recycled proteins) were added to the plates, in addition to serial dilutions from 0.122 to 250 ng/mL of the proteins tested diluted in PBST-M, which were used as standards to quantify protein levels. Following a 2 h incubation period at room temperature, a goat anti-human Fc polyclonal antibody conjugated to alkaline phosphatase (Sigma-Aldrich, A9544) diluted 1:5,000 in PBST-M was added and incubated for 1 h at room temperature. The ELISA was developed, and absorbance was measured as indicated above.

HERA experiments were independent and numerical data were summarized as the mean ± SD using GraphPad Prism9 software (San Diego, CA). Each global mean was compared using an unpaired Student’s *t*-test. Two-tailed *p*-values ≤ 0.05 were considered statistically significant.

### In Vitro Blocking ELISA

*In vitro* neutralization of the α-cbtx interaction with α7-AChR by Quads was performed using a similar ELISA protocol to that described above but with some modifications. Briefly, high-binding 96-well plates (Corning) were coated overnight at 4 °C with 100 ng of α7-AChR/well. With three washes in between each subsequent step, using PBST (PBS + 0.1% Tween 20), and incubation at room temperature for 1 h, the coated ELISA plates were blocked with 1% BSA. Next, mixtures of serially diluted anti-α-cbtx Quads starting at 5 μg/mL and a fixed amount of biotinylated α-cbtx (0.0175 μg/mL) were preincubated at room temperature for 30 min prior to being added to the coated plates. Wells containing only the biotinylated α-cbtx with no added anti-α-cbtx Quad or wells containing blocking buffer only (0.1% BSA in PBST) were used as controls to determine the percentage α-cbtx α7-AChR binding inhibition. Free α-cbtx bound to α7-AChR was detected using HRP-conjugated streptavidin (Abcam, 1 in 15,000 dilution in PBST). The ELISA signal was measured as described above in the indirect ELISA. Each concentration was run in duplicate and presented as mean ± SD values.

### *In Vitro* Cross-Neutralization of Long α-Neurotoxins Using an Automated Patch Clamp

Planar whole-cell patch-clamp experiments were carried out on a Qube 384 automated electrophysiology platform (Sophion Bioscience), where 384-channel patch chips with 10 parallel patch holes per channel (patch hole diameter ∼1 μm, resistance 2.00 ± 0.02 MΩ) were used.

A human-derived rhabdomyosarcoma RD cell line (CCL-136, from ATCC) endogenously expressing the muscle-type nicotinic acetylcholine receptors (nAChR) composed of the α1, β1, δ, γ, and ε subunits was used. The cells were cultured according to the manufacturer’s guidelines. On the day of the experiment, cells were enzymatically detached from the culture flask and brought into suspension. For patching, the extracellular solution contained 145 mM NaCl, 10 mM HEPES, 4 mM KCl, 1 mM MgCl_2_, 2 mM CaCl_2_, and 10 mM glucose, pH adjusted to 7.4, and osmolality adjusted to 296 mOsm, while the intracellular solution contained 140 mM CsF, 10 mM HEPES, 10 mM NaCl, 10 mM EGTA, pH adjusted to 7.3, and osmolality adjusted to 290 mOsm.

In the experiments, an nAChR-mediated current was elicited by 70 μM acetylcholine (ACh, Sigma-Aldrich), approximately the EC_80_ value, and after compound wash-out, 2 U acetylcholinesterase (Sigma-Aldrich) was added to ensure complete ACh removal. A second ACh addition was used to evaluate the effect of the toxin (app. IC_80_ value; α-cbtx 1.5 nM; α-eptx 0.81 nM; α-bgtx 6.4 nM; Nm8 14.0 nM) in combination with 3 nM of Quad. Toxins and Quads were prepared in extracellular solution supplemented with 0.1% BSA and co-incubated for at least 30 min before application, and the patched cells were preincubated with the toxin and Quad mixture for 5 min prior to the second ACh addition. Measurements were performed in quadruplicate, and the error is reported as the mean ± SD. The inhibitory effect of the toxins was normalized to the full ACh response and averaged in the group. The data analysis was performed in a Sophion analyzer (Sophion Bioscience).

## Supporting Information Available

FIDA complex sizes and affinities; ITC thermodynamic parameters; ELISA binding; SEC retention volumes; percentage areas; and calculated molecular weights and DLS (Tables S1–S6) (PDF)
