# Supplementary material for: Generation of Multivalent Nanobody-Based Proteins with Improved Neutralization of Long α-Neurotoxins from Elapid Snakes
Source: Bioconjug Chem. 2022 Jul 23;33(8):1494–504. doi: 10.1021/acs.bioconjchem.2c00220 (PMC9389527; doi:10.1021/acs.bioconjchem.2c00220)
Supplement: Supplementary file 1 — bc2c00220_si_001.pdf [file bc2c00220_si_001.pdf]

Supporting information

Generation of multivalent nanobody-based proteins with improved neutralization of long α-neurotoxins from elapid snakes

Jack Wade[1,†], Charlotte Rimbault[1,†], Hanif Ali[2], Line Ledsgaard[1], Esperanza Rivera-de-Torre[1], Maher Abou Hachem[1], Kim Boddum[4], Nadia Mirza[3], Markus-Frederik Bohn[1], Siri A. Sakya[5,6], Fulgencio Ruso-Julve[5,6], Jan Terje Andersen[5,6], Andreas H. Laustsen[1]**

[1]Department of Biotechnology and Biomedicine, Technical University of Denmark, DK-2800 Kongens Lyngby, Denmark

[2]Quadrucept Bio Ltd, Kemp House, 152 City Road, London, EC1V 2NX, United Kingdom

[3]Fida Biosystems ApS, DK-2860 Søborg, Copenhagen, Denmark

[4]Sophion Bioscience, DK-2750 Ballerup, Denmark

[5] Department of Immunology, Oslo University Hospital Rikshospitalet, N-0372, Oslo, Norway.

[6]Department of Pharmacology, Institute of Clinical Medicine, University of Oslo, N-0372, Oslo,

Norway

*Correspondence:

Andreas H. Laustsen

ahola@bio.dtu.dk

Fulgencio Ruso-Julve

fulgencr@medisin.uio.no

[†]These authors contributed equally to this work.

Table S1: Molecular weight of nanobody and Quad proteins. Molecular weights were calculated using the ProtParam tool from Expasy (https://web.expasy.org/protparam/).

Molecule	Valence	MW monomer (kDa)	MW tetramer (kDa)
V_HH	1	15	
Q187	4	19.6	78.3
Q188	8	33.2	132.7
Q189	8	33.4	133.6
Q190	4	46.7	186.8
Q191	8	60.3	241.2
Q193	4	22.8	91.2
Q194	8	36.4	145.6
Q207/208	16	44.3 + 39.3 (LC)	334.4

Table S2: FIDA results for nanobody and Quad proteins binding to labeled long α-neurotoxins. The excess indicator model from the FIDA One software was used to fit the measurements from a single experiment performed with technical duplicates.

Molecule	Indicator	Indicator Rh (nm)	Complex Rh (nm)	K_D (nM)	R^2
V_HH	α-cbtx	1.7	2.7	25.2	0.99
Q187	α-cbtx	1.4	4.8	2.5	0.98
Q188	α-cbtx	1.5	5.4	1.9	0.99
Q189	α-cbtx	1.4	5.1	1.6	0.98
Q190	α-cbtx	1.4	7.8	1.3	0.98
Q191	α-cbtx	1.3	7.4	0.8	0.98
Q193	α-cbtx	1.7	5.1	3.3	0.99
Q194	α-cbtx	1.8	6.8	2.2	0.99
Q207/208	α-cbtx	1.6	9.1	0.7	0.99
Q187	Nm8	1.3	4.6	14.4	0.98

Table S3: DLS measurements of select Quad molecules. Measurements of the monodispersity were performed at a concentration of 1 mg/mL in triplicate using a Zetasizer Nano instrument.

Molecule	Polydispersity (%)	Estimated Dh (nm)
Q189	3.6	13.00 ± 3.87
Q193	2.1	13.50 ± 4.58
Q194	9.5	17.12 ± 6.25

Table S4: Indirect ELISA results of nanobody and Quad proteins binding to immobilized α-cobratoxin. The titration-ELISA data are the results of two independent experiments and the equation described in Li et al. 2011 has been fitted to those data using GraphPad Prism.

Molecule	OD_{min} (405 nm)	OD_{max} (405 nm)	K_D (nM)	R^2
V_HH	0.1134	2.648	0.03898	0.99
Q187	0.2286	2.647	0.006322	0.99
Q188	0.1345	2.654	0.008281	0.99
Q189	0.2022	2.637	0.005865	0.99
Q190	0.1511	2.657	0.00552	0.99
Q191	0.1139	2.665	0.005007	0.99
Q193	0.2988	2.647	0.005462	0.99
Q194	0.2103	2.652	0.004387	0.99
Q207/208	0.09299	2.67	0.007629	0.99

Table S5: ITC binding parameters of nanobody and select Quad proteins. Values of association constants (K_A), binding enthalpy (ΔH), and binding stoichiometry (N) obtained in ITC analysis are expressed as means ± SD of duplicate experiments. Dissociation constants (K_D) were calculated from the reciprocal of K_A. The Gibbs free energy change (ΔG^0) and the entropy change (ΔS^0) were calculated from the equations $\Delta G^0 = -R \cdot T \cdot \ln(K_A)$ and $T \cdot \Delta S^0 = \Delta H - \Delta G^0$, respectively (R, gas constant; T, absolute temperature).

Molecule	K_A ($\times 10^7$ M^{-1})	K_D ($\times 10^{-9}$ M)	ΔG^0 (kcal mol^{-1})	ΔH (kcal mol^{-1})	ΔS^0 (cal K^{-1} mol^{-1})	N (sites)
V_HH	19.5 ± 4.6	5.1	-11.3	-18.83 ± 0.24	-25.2	0.92 ± 0.22
Q187	13.26 ± 9.7	7.5	-11.1	-18.08 ± 0.25	-23.4	3.84 ± 0.07
Q189	11.52 ± 4.9	8.68	-11	-16.65 ± 0.07	-18.9	8.03 ± 1.05
Q207/208	10.7 ± 0.7	9.35	-10.9	-17.65 ± 0.21	-22.6	13.35 ± 1.20

Table S6: SEC analysis of Quad molecules. Quads were loaded on a HiLoad Superdex200 Increase 10/300 GL column with PBS as an eluent. Characteristics of the main elution peaks are in bold. The Molecular Mass (kDa) is the molecular weight determined for the observed elution volumes after calibration of the column with the Protein Standard mix 15-600 kDa from Sigma-Aldrich.

**Molecule**	**Peak number**	**Retention volume (mL)**	**Relative area (%)**	**Molecular Mass (kDa)**
V_HH	1	17.56	100	15.2
Q187	**1**	**13.15**	**96.87**	**90.4**
	2	17.31	3.01	15.3
Q188	**1**	**11.48**	**96.94**	**182**
	2	16.30	2.39	25.4
Q189	**1**	**11.90**	**94.91**	**153.7**
	2	18.18	4.34	11.8
Q190	1	9.06	11.25	490.1
	**2**	**10.09**	**88.51**	**321.1**
Q191	1	9.56	97.97	418.8
	2	16.29	1.43	23.7
Q193	1	9.42	4.90	444.8
	2	10.16	11.54	324.4
	**3**	**11.75**	**81.30**	**164.6**
	4	17.57	1.22	13.7
Q194	1	9.39	11.3	428.4
	**2**	**10.65**	**86.26**	**255.6**
	3	16.26	2.22	25.9
Q207/208	**1**	**9.17**	**100**	**496**
